# Natural Killer Cells and Liver Fibrosis

**DOI:** 10.3389/fimmu.2016.00019

**Published:** 2016-01-29

**Authors:** Frank Fasbender, Agata Widera, Jan G. Hengstler, Carsten Watzl

**Affiliations:** ^1^Department for Immunology, Leibniz Research Centre for Working Environment and Human Factors (IfADo), Technische Universität Dortmund, Dortmund, Germany; ^2^Department for Toxicology, Leibniz Research Centre for Working Environment and Human Factors (IfADo), Technische Universität Dortmund, Dortmund, Germany

**Keywords:** natural killer cells, liver disease, fibrosis, stellate cells

## Abstract

In the 40 years since the discovery of natural killer (NK) cells, it has been well established that these innate lymphocytes are important for early and effective immune responses against transformed cells and infections with different pathogens. In addition to these classical functions of NK cells, we now know that they are part of a larger family of innate lymphoid cells and that they can even mediate memory-like responses. Additionally, tissue-resident NK cells with distinct phenotypical and functional characteristics have been identified. Here, we focus on the phenotype of different NK cell subpopulations that can be found in the liver and summarize the current knowledge about the functional role of these cells with a special emphasis on liver fibrosis. NK cell cytotoxicity can contribute to liver damage in different forms of liver disease. However, NK cells can limit liver fibrosis by killing hepatic stellate cell-derived myofibroblasts, which play a key role in this pathogenic process. Therefore, liver NK cells need to be tightly regulated in order to balance these beneficial and pathological effects.

## Introduction

Natural killer (NK) cells are innate lymphoid cells (ILC) that can kill virus-infected or transformed cells. Additionally, they regulate adaptive immune responses *via* contact-dependent signals and the secretion of cytokines (1). NK cell cytotoxicity is regulated by activating and inhibitory surface receptors and is additionally modulated by cytokines (2). Inhibitory NK cell receptors include killer cell Ig-like receptors (KIR) in humans and Ly49 family members in mice, both of which interact with MHC I to ensure the self-tolerance against healthy cells. NK cell activation can be mediated by a variety of different surface receptors, such as NKG2D, NKp46, and NKp30 (3). Initially, human NK cells have been divided into two functionally distinct subpopulations based on the expression level of CD56. In recent years, more subpopulations of NK cells have been identified, and we now know that in addition to conventional circulating NK cells, there are also tissue-resident NK cells with distinct phenotypical and functional characteristics (4). Here, we summarize the current knowledge about NK cells in the liver and focus on the role of these immune cells in liver fibrosis.

## NK Cells in the Liver

The liver mainly consists of hepatocytes, which make up approximately 80% of liver cells. Non-hepatocytes include about 20% lymphocytes, 20% Kupffer cells, 40% endothelial cells, 20% stellate cells, and biliary cells (5). NK cells in the liver were first described by electron microscopy of rat liver and initially named “pit cells” (6). They reside in liver sinusoids and can make up to 50% of the liver lymphocyte population in humans (7, 8). This is in contrast to the frequency of NK cells in peripheral blood, where they only account for 5–15% of lymphocytes. It remains unclear what regulates this enrichment of NK cells in the liver. It is believed that cell-to-cell and cell-to-matrix interactions play an important role in this process (9). For example, NK cell infiltration in the liver can be blocked by neutralizing antibodies against CD2, CD11a, CD18, and ICAM-1 (CD54) (10), suggesting that adhesion to sinusoidal endothelial cells is an important step in their recruitment. Endothelial cells also express vascular adhesion protein-1 (VAP-1) (11), which can be recognized by Siglec-9 and could represent another mechanism of liver NK cell enrichment (12).

Liver NK cells have been extensively compared to peripheral blood NK cells and differ in activation level, cytotoxicity, and maturation (13). In general, liver NK cells are more activated as they express high levels of the activation marker CD69, more perforin, and granzyme B (8, 14–17). As a consequence, they show higher cytotoxicity compared to peripheral blood NK cells. However, they are also less mature compared to peripheral blood NK cells (15, 16, 18, 19).

**Figure 1:**
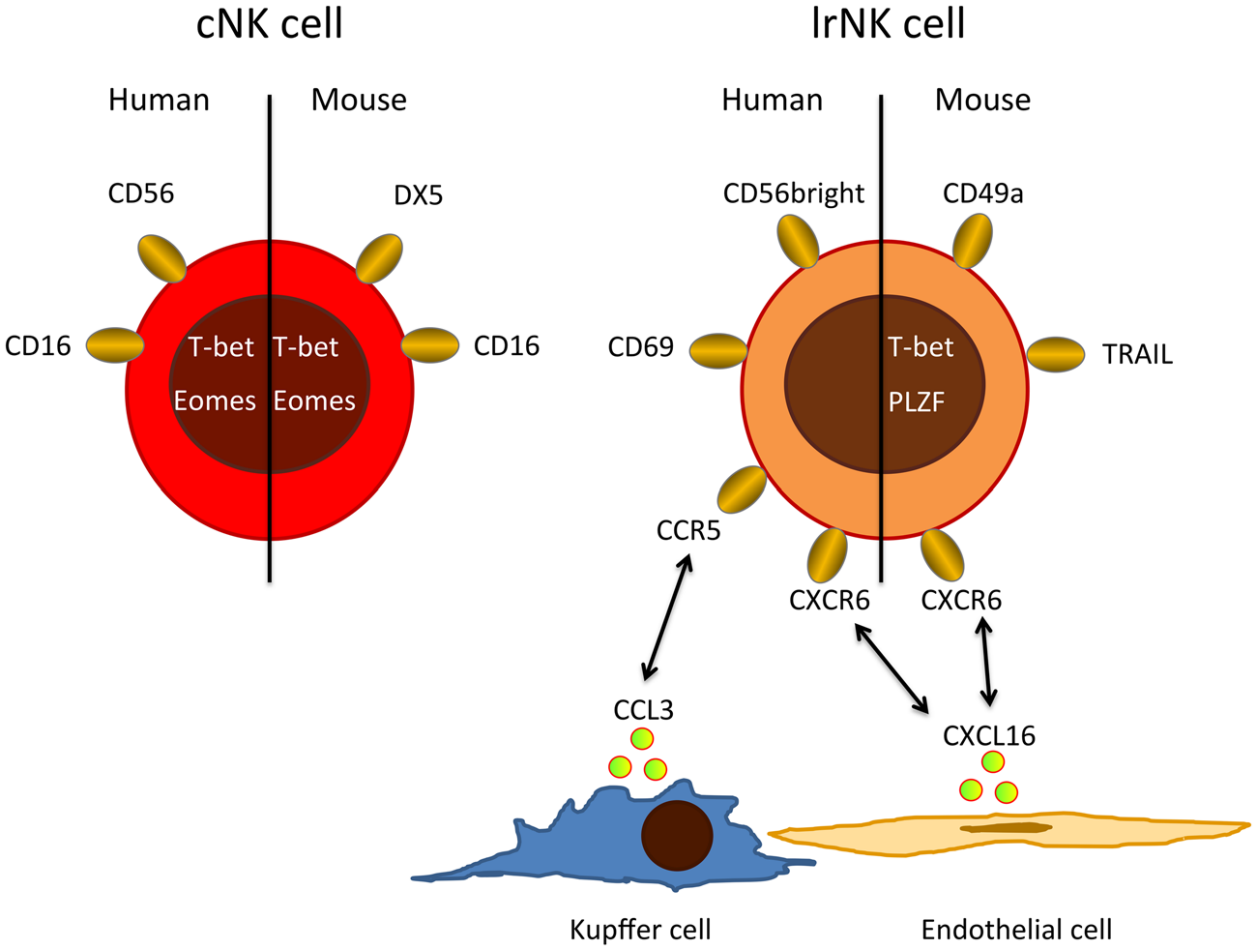
**Major phenotypic differences between cNKs and lrNKs**. Human cNKs are mostly CD56dim and express CD16, whereas lrNKs show a CD56bright phenotype and are negative for CD16, but express homing markers, such as CXCR6 and CCR5. Possible ligands for these homing-associated receptors are expressed by endothelial cells, Kupffer cells, and circulating cNKs. In mice, lrNKs are CD49a^+^DX5^−^ and depend on the transcription factors T-bet and PLZF, while cNK are CD49^−^DX5^+^ and need the transcription factor Eomes for their development.

In humans, NK cells are grouped into CD56^dim^ and CD56^bright^ cells with CD56^dim^ NK cells accounting for up to 90% of all NK cells in peripheral blood and spleen. In contrast, equal numbers of CD56^dim^ and CD56^bright^ NK cells are found in the liver (16, 20). The CD56^dim^ NK cell population in the liver seems to resemble circulating conventional NK cells (cNKs). However, recent evidence suggests that liver CD56^bright^ NK cells differ from cNK and represent a distinct, liver-resident NK cell (lrNK) population dependent on the chemokine receptor CXCR6 (Figure 1) (20). lrNKs show increased expression of CD69 and the homing markers CXCR6 and CCR5. Engagement of these receptors by CXCL16 from hepatic sinusoidal endothelial cells (21) and CCL3 from Kupffer cells as well as CCL5 from T and NK cells, respectively, retains lrNK cells in a unique chemokine environment. The development and differentiation of lrNK cells is incompletely understood. Cells corresponding to all described developmental intermediates of NK cells have been identified in the adult human liver (16), indicating that NK cell precursors are recruited from peripheral blood and that lrNK cells may differentiate in the liver.

Conventional NK and lrNK cells have also been identified in mice (Figure 1), where NK cells make up only 5–10% of the liver lymphocytes. About half of these murine liver NK cells resemble cNKs, but they are DX5^−^ and express high levels of TRAIL (22–24). In mice, there is clear evidence that cNK and lrNK originate from different developmental programs. lrNK rely on the transcription factors T-bet and PLZF for their development (25, 26), but they are independent of Eomes (27), which is critical for the development of cNKs (28–30). Mouse lrNK are CD49a^+^, DX5^−^ and show expression of homing markers (13). They are similar in their phenotype and development to mucosal group 1 innate lymphoid cells (ILC1) (19).

Interestingly, a hepatic NK cell population has been reported that can display adaptive-like immune memory against haptens or viral antigens (31). This antigen-specific type of NK cell memory is confined to CXCR6-positive hepatic NK cells, which were identified as the CD49a^+^, DX5^−^ lrNK population (32). Recently, a human intrahepatic CD49a^+^ NK cell population was identified that was not detectable in afferent or efferent hepatic venous or peripheral blood (33). These NK cells express KIR and NKG2C, indicative of having undergone clonal-like expansion. They are CD56^bright^ and express low levels of CD16, CD57, and perforin. Because this population was only detected at low frequencies (2.3% of hepatic NK cells) and not in every donor, it might represent a subpopulation of lrNKs. It is interesting to speculate that these cells can also mediate certain kinds of adaptive memory (3). In support of this, antigen-specific memory of hepatic NK cells has recently been shown in macaques following infection and vaccination (34).

The fact that NK cells represent the major lymphocyte population in human liver suggests relevant functions. Indeed, liver NK cells have been shown to influence many physiological and pathophysiological processes, such as viral infections, liver tumorigenesis, liver injury, and inflammation (13). In the following section, we will focus on their role in liver fibrosis.

## Liver Fibrosis and NK Cells

An outstanding feature of the liver is its enormous regeneration capacity that has evolved to protect animals from liver loss by hepatotoxic plants (35, 36). Acute destruction of more than 50% of the liver tissue can be regenerated within a relatively short period of time leading to the perfect restoration of tissue architecture and function (37–39). However, repeated destruction of hepatocytes leads to scar formation and fibrosis (40). Fibrosis is characterized by excess extracellular matrix, which initially compromises liver function only to a minor degree (41). However, fibrosis may progress to cirrhosis, where normal liver architecture is replaced by nodules of hepatocytes surrounded by wide streets of fibrotic tissues, which massively constrict blood flow and reduce liver function. Fibrosis and cirrhosis can, in principle, be caused by any condition that repeatedly kills a critical fraction of hepatocytes, such as alcohol abuse, repeated administration of hepatotoxic drugs, viral hepatitis, cholestatic disorders, or hereditary metabolic liver diseases (42, 43).

**Figure 2:**
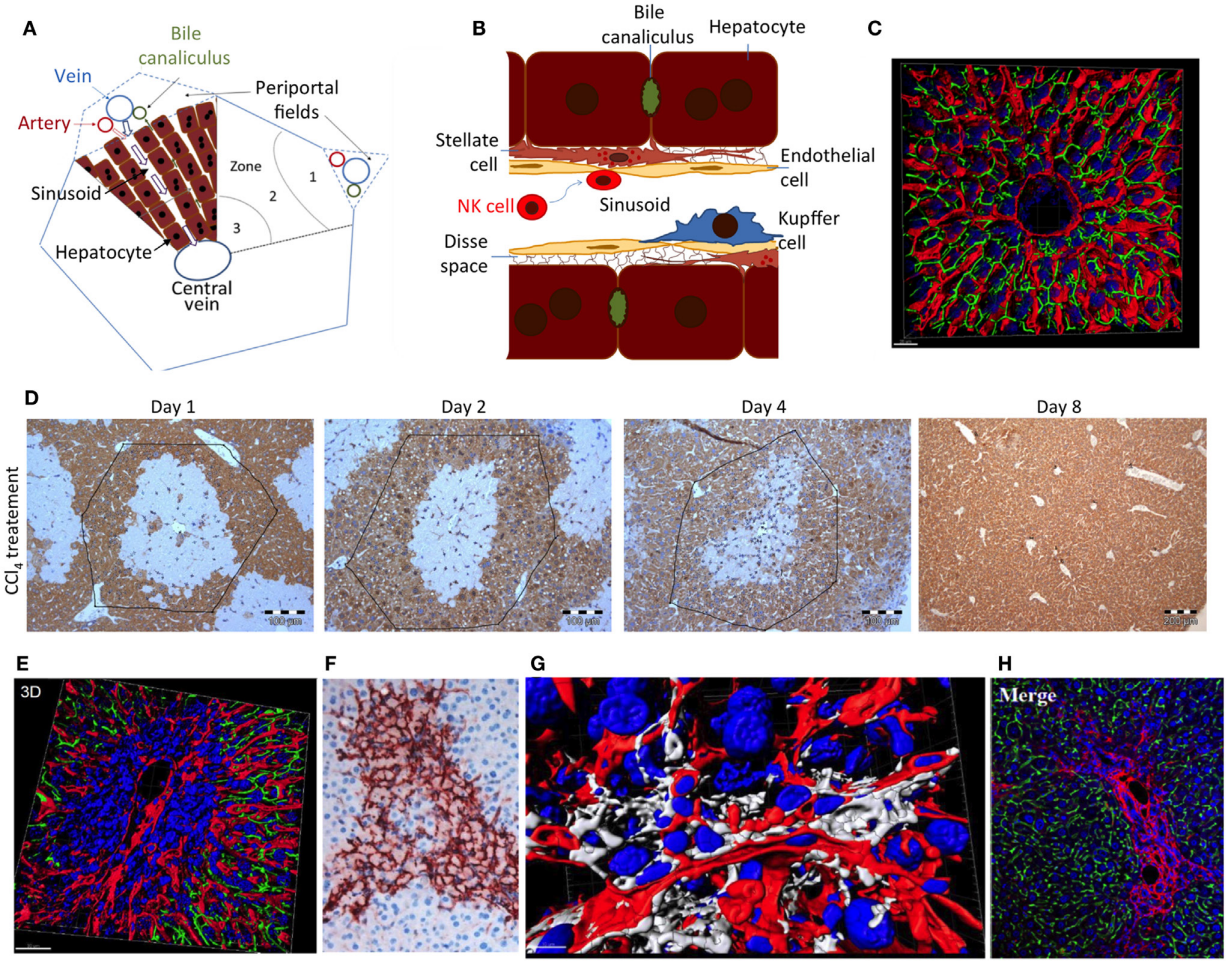
**(A)** Schedule of a liver lobule and **(B)** sinusoid; **(C)** reconstructed liver lobule showing the sinusoidal endothelial cells (LSEC) in red, bile canaliculi in green, and nuclei in blue. **(D)** Damage and regeneration process after administration of a hepatotoxic dose of CCl_4_ to mice. The initial pericentral damage (day 1) is completely regenerated until day 8 after intoxication. **(E)** Necrotic area 2 days after CCl_4_ administration, illustrating a pericentral nuclear dense region with compromised microvessels. **(F)** Alpha-smooth muscle actin staining of activated HSC in a necrotic region 2 days after CCl_4_ administration. **(G)** Reconstruction of a necrotic region 2 days after CCl_4_ administration, visualizing stellate cells in white, LSEC in red, and nuclei in blue. **(H)** Collagen staining to visualize a fibrotic street after repeated CCl_4_ doses. Imaging was performed with livers of male C57BL6/N mice as described in Ref. (48); **(D)** from Ref. (37).

Hepatic stellate cells (HSCs) play a key role in pathogenesis of liver fibrosis (44–46). They were described by Karl Wilhelm von Kupffer in 1876, but their role in liver disease was only identified in the 1980s (47). HSCs are located between hepatocytes and the endothelial cells of the sinusoids in the 0.2–1-μm wide extracellular matrix-filled Disse space (Figure 2) (48). Activation of HSCs and transdifferentiation to myofibroblasts, the major extracellular matrix-producing cell in fibrotic liver, represents a critical step on the path to fibrosis. Typically, cell death of hepatocytes creates an inflammatory microenvironment, which activates HSCs. Key factors driving HSC activation are transforming growth factor beta1 (TGFbeta1) and platelet-derived growth factor (PDGF) family members. Moreover, numerous cytokines and chemokines released by infiltrating immune cells modify this process (42, 44, 49). Finally, this leads to a situation where extracellular matrix formation by activated HSCs outbalances the mechanisms of collagen degradation by matrix metalloproteases.

The molecular mechanisms of liver fibrosis and HSC activation have been reviewed comprehensively (40, 42, 43). The present article focuses on a specific mechanism, which antagonizes fibrosis formation, namely NK cell-mediated cytotoxic activity against HSC-derived myofibroblasts, which has been described in numerous articles since its first descriptions in 2006 (50, 51).

To study interactions between NK cells and HSCs as well as their role for liver fibrosis, the most frequently applied experimental tools are the mouse models of liver damage and cultivated human and rodent HSC. In mice, liver fibrosis can be induced by repeated administration of hepatotoxic compounds, such as CCl_4_. In this experimental scenario, additional interventions can be performed, such as the elimination of NK cells by antibodies and the genetic deletion of specific receptors and ligands. Alternatively, HSCs can be isolated from liver tissue and brought into culture, where they spontaneously differentiate to alpha-SMA-positive myofibroblasts, which can be tested in killing assays with NK cells. Based on such experimental models, we now have a detailed picture of the interaction between NK cells and HSCs.

In general, the activation of HSCs in response to hepatocyte damage results in changes that increase NK cell stimulation and decrease NK cell inhibition. A key mechanism is that early-activated HSCs produce increased amounts of retinoic acid, which leads to elevated expression of RAE-1 (52). RAE-1 is a ligand for the activating NK cell receptor NKG2D and together with MICA (53) triggers killing of activated HSCs by NK cells. Human and mouse HSCs additionally express a ligand for the activating NKp46 receptor. This also causes HSC killing by NK cells, which ameliorates liver fibrosis (54). Recent data suggest a role of the activating receptor NKp30 in this process (55). In addition to the increased activation of NK cells, reduced inhibition also plays a role in the cytotoxic attack of NK cells against HSCs. Upon activation of HSCs, MHC class I is downregulated, resulting in the reduced engagement of inhibitory NK cell receptors and enhanced killing (50, 56). Experimentally, reducing inhibitory Ly49 receptor expression on mouse NK cells by siRNA-mediated silencing, therefore, increases HSC killing by NK cells and ameliorates liver fibrosis. Inflammatory cytokines can further influence this process. NK cell-derived IFN-γ has antifibrotic effects by inducing HSC apoptosis and cell cycle arrest (57, 58). However, clinical trials with IFN-γ led to disappointing results, and it has been reported that HSC-specific delivery is critical for its antifibrotic effect (59). IFNα has been shown to increase expression of TRAIL on the surface of NK cells (60, 61). Simultaneously, activation of HSCs leads to increased expression of the TRAIL receptor on the HSC surface, resulting in enhanced NK cell-mediated HSC killing. Recombinant expression of human TRAIL on HSCs has been shown to induce HSC apoptosis and blocking TRAIL by antibodies antagonized this effect (62). In contrast, TGF-β levels are elevated during chronic liver injury and suppress the antifibrotic function of NK cells through downregulation of NKG2D and 2B4 surface expression (63–65).

While the mechanisms mentioned above give some insight into the molecular mechanisms about how NK cells antagonize fibrosis, open questions remain. It is unclear which population of liver NK cells is responsible for limiting fibrosis. lrNK may already be in place to directly interact with HSCs. However, the role of lrNK cells in liver fibrosis has not been addressed in detail. *In vitro* experiments mostly use NK cells from human peripheral blood or mouse spleen to study the killing of HSCs. For *in vivo* depletion experiments using antibodies, it is unclear if the tissue-resident lrNK cells can be efficiently depleted. Therefore, it remains elusive what role lrNK cells play during fibrosis.

Hepatic stellate cells play an important role during the regeneration of liver damage, when they infiltrate the wound and by secretion of cytokines, such as HGF, help to orchestrate the regeneration process (66). However, as soon as hepatocytes have repopulated the dead cell area of the wound, the further presence of activated HSCs is deleterious and acts profibrotic. It is therefore important that NK cells remove activated HSCs after they have accomplished their mission in regeneration. Indeed, *in vivo* and *in vitro* experiments show that NK cells kill early activated, but not quiescent or fully activated, HSCs (52, 67). This may be due to an increase of the ratio of activating versus inhibiting mechanisms in early activated HSCs (62). However, so far little attention has been paid to the massive architectural changes during liver damage and regeneration, which may impact NK cell/HSC interactions. In the healthy liver, NK cells float in the sinusoidal blood or roll along sinusoids (Figures 2A,B). In this situation, HSCs are shielded from NK cells by endothelial cells (LSEC) (Figure 2B). Cytotoxic T cells may overcome the endothelial barrier and probe antigens on subsinusoidal cells by extending cytoplasmic protrusions through sinusoidal endothelial fenestrae (68). However, any shielding function of LSEC is transiently lost during liver damage induction and regeneration (Figures 2D–G). Most hepatotoxic compounds that require activation by cytochrome P450 enzymes induce necrosis in the center of liver lobules, visible as a pale region in Figure 2D. Numerous immune cells infiltrate the necrotic area leading to high nuclear density (Figure 2E). Additionally, activated alpha-smooth muscle actin-positive HSCs form an alveolar scaffold in the necrotic area between days 2 and 4 after damage induction (Figure 2F). In this period, activated HSCs are no longer shielded from immune cells by endothelial cells. With their long delicate protrusions, they get into direct contact with several types of immune cells (Figure 2G). Only 8 days after damage induction, all activated HSCs disappear from the tissue. It has not yet been studied whether the direct accessibility of HSCs in damaged regions of the liver to NK and other immune cells is the reason for their abrupt disappearance. Additionally, in liver fibrosis, activated HSCs persist for longer periods. In this situation, they are immured by collagen fibers (Figure 2H), which may prevent access of NK cells to their target.

The interaction between NK cells and HSCs is additionally regulated by other cells and processes. Kupffer cells and dendritic cells can enhance NK cell activation under immune stimulatory conditions, such as Toll-like receptor stimulation or viral liver disease (69–71). Regulatory T cells can inhibit NK cell activity and thereby limit their antifibrotic function during viral hepatitis (72, 73).

While NK cell activity may be beneficial for the regulation of liver fibrosis, it can also have negative effects. Indeed, very similar molecular mechanisms by which activated HSCs are removed have also been described for NK cell-mediated killing of hepatocytes. Importantly, RAE-1, MICA/B, B7-H6, TRAIL-receptor, and Fas on hepatocytes, as well as NKG2D, NKp30, and TRAIL ligand on NK cells have been reported to play a role in NK cell-induced hepatocyte death (14, 23, 55, 74–77). This illustrates that NK cell-activating therapeutic strategies (78, 79) should be considered with care, since the tightly controlled mechanisms of selectively killing-activated HSC may easily switch to a situation where also hepatocytes become targeted, which would promote liver damage and aggravate the profibrotic pressure.

When studying the function of NK cells in liver, it is important to mention a potential experimental pitfall. A well-studied example is the misinterpretation of experimental data that NK and NKT cells enhance acetaminophen (APAP)-induced liver damage in mice (80). Since others could not reproduce the result, the experiments have been carefully revisited (81). It was shown that the DMSO used in the original study to dissolve the APAP has adjuvant-like functions and stimulates the activity of NK and NKT cells to enhance APAP-induced liver damage. Interestingly, a solvent control would not be sufficient to avoid this misinterpretation as the adjuvant function of DMSO does not result in liver damage alone, but only in combination with APAP. This has to be taken into account when investigating the role of NK cells in drug-induced liver damage.

## Outlook

Natural killer cell cytotoxicity limits HSC-mediated liver fibrosis, and in recent years, we have learned much about the molecular details of this interaction. However, careful *in vivo* analysis will still need to address several important questions, such as the spatiotemporal details of NK–HSC interaction and the role of the different subpopulations of liver NK cells in this process. Additionally, NK cells are part of a larger group of ILC. ILCs are mostly tissue-resident cells with important functions in tissue homeostasis and immunity against pathogens. There are first indications that these novel immune cells can also influence the process of liver fibrosis (82, 83), which may lead to an exciting research field in the future.

## Author Contributions

All authors participated in writing the manuscript.

## Conflict of Interest Statement

The authors declare that the research was conducted in the absence of any commercial or financial relationships that could be construed as a potential conflict of interest.
